# Evidence-based ethics? On evidence-based practice and the "empirical turn" from normative bioethics

**DOI:** 10.1186/1472-6939-6-11

**Published:** 2005-11-08

**Authors:** Maya J Goldenberg

**Affiliations:** 1 Department of Philosophy, Michigan State University, 503 South Kedzie Hall, East Lansing, Michigan, USA

## Abstract

**Background:**

The increase in empirical methods of research in bioethics over the last two decades is typically perceived as a welcomed broadening of the discipline, with increased integration of social and life scientists into the field and ethics consultants into the clinical setting, however it also represents a loss of confidence in the typical normative and analytic methods of bioethics.

**Discussion:**

The recent incipiency of "Evidence-Based Ethics" attests to this phenomenon and should be rejected as a solution to the current ambivalence toward the normative resolution of moral problems in a pluralistic society. While "evidence-based" is typically read in medicine and other life and social sciences as the empirically-adequate standard of reasonable practice and a means for increasing certainty, I propose that the evidence-based movement in fact gains consensus by *displacing *normative discourse with aggregate or statistically-derived empirical evidence as the "bottom line". Therefore, along with wavering on the fact/value distinction, evidence-based ethics threatens bioethics' normative mandate. The appeal of the evidence-based approach is that it offers a means of negotiating the demands of moral pluralism. Rather than appealing to explicit values that are likely not shared by all, "the evidence" is proposed to adjudicate between competing claims. Quantified measures are notably more "neutral" and democratic than liberal markers like "species normal functioning". Yet the positivist notion that claims stand or fall in light of the evidence is untenable; furthermore, the legacy of positivism entails the quieting of empirically non-verifiable (or at least non-falsifiable) considerations *like *moral claims and judgments. As a result, evidence-based ethics proposes to operate with the implicit normativity that accompanies the production and presentation of *all *biomedical and scientific facts unchecked.

**Summary:**

The "empirical turn" in bioethics signals a need for reconsideration of the methods used for moral evaluation and resolution, however the options should not include obscuring normative content by seemingly neutral technical measure.

## Background

The increase in empirical methods of research in bioethics (or "empirical ethics") over the last two decades is typically perceived as a welcomed broadening of the discipline, with increased integration of social and life scientists into the field and ethics consultants into the clinical setting [1]. Evidence-based ethics is the newest empirical approach to bioethics inquiry, and while this method is still underdeveloped and the growing body of literature still small, there is good reason to expect this approach to really "take off" given the currency of "evidence based" approaches in so many professional disciplines. This paper is an effort to temper the momentum of the evidence-based movement and to reject its proliferation into bioethics. By examining the norms and implications of evidence-based practice in medicine, I aim to demonstrate that an evidence-based approach is incompatible with bioethics' normative mandate and therefore evidence-based ethics should not be pursued.

### Empirical research in bioethics

Empirical research in bioethics (or "empirical ethics") is "the application of research methods in the social sciences (such as anthropology, epidemiology, psychology, and sociology) to the direct examination of issues in [bioethics]" [2]. Empirical approaches describe (rather than prescribe) "particular state[s] of affairs that [have] some moral or ethical relevance" [2] and are thought to enrich bioethics by calling attention to the social, cultural, and cross-cultural aspects of morality accessed via the opinions, interests and beliefs of patients, families, physicians, nurses and others involved in care-giving [3]. For example, empirical research can help describe cultural beliefs about the appropriateness of informing the patient of a diagnosed life-threatening illness, which will inform deliberation about the extent to which it is morally important for clinicians to provide comprehensive information to patients in different cultural contexts [4]. Similarly, empirical research can delineate popular attitudes and experiences related to contentious issues such as abortion, cloning, stem-cell research, and physician-assisted suicide for consideration in discussions and policy formulations [4]. Empirical work can also map the effects of particular interventions aimed at improving how clinicians or policy makers attempt to meet ethical obligations, such as whether a particular method of presenting health related information to a patient actually improves the patient's understanding of her circumstances and the quality of informed consent [4].

It is likely because proponents of empirical approaches to bioethics focus on differentiating this area of bioethics from normative ethics that the literature tends to use such loose overarching descriptors as "an amalgam of empirical contributions" [1] and "methodological roots in social sciences...to gather quantitative and qualitative data about ethical issues" [1] when characterising empirical ethics. The ease at which empirical ethics is generalised, and the differences between the various social scientific disciplines represented under this heading glossed over, may also be assisted by the common presumption that empirical research presents "only the facts". This understanding leads to underappreciation of the values typical to each discipline as well as the beliefs of the individual practitioner that influence the data gathering and interpretation [5]. While presumably no one would deny the different orientations, research agendas, and methods, of, say, psychology and sociology, the emphasis placed on similarities or shared features in order explain the novelty of empirical ethics and distinguish it from philosophical approaches to bioethics takes important attention away from the relevant differences between the social scientific disciplines that may render some empirical approaches incompatible with the goals of ethical inquiry. This paper focuses on one method of empirical ethics – evidence-based bioethics, which is grounded in clinical epidemiology and supported by the discipline's most distinctive application, evidence-based medicine.

## Discussion

### Evidence based medicine and the evidence-based movement

While evidence-based ethics arises within the momentum of what has been called "the empirical turn in bioethics" [1] – the increased interest in empirical research in bioethics – it draws unique content from the evidence-based movement that began in medicine only a decade and a half ago in the form of "evidence-based medicine" and then exploded into other professional disciplines. The term "evidence based medicine" was first introduced in a ubiquitous 1992 publication of the *Journal of the American Medical Association *as a "new paradigm" in medical education and practice by a group of professors of clinical epidemiology, medical informatics, and biostatistics at McMaster University calling themselves "The Evidence Based Medicine Working Group" [6]. The evidence-based medicine movement diagnosed the problems of medical error and wasteful healthcare spending as stemming from the prevalent use of unestablished medical interventions and proposed to remedy these difficulties by way of a decision-making technology that would eschew unsystematic and so-called "intuitive" methods of individual clinical experience in favour of a more scientifically rigorous approach. According to the dictates of evidence-based decision-making, clinical decisions should be based on the best available scientific evidence and "identifying the best evidence means using epidemiological and biostatistical ways of thinking" [7]. The methodological privileging of outcomes measures, statistical analysis, and indexes of aggregate behaviour that characterises clinical epidemiology serves to distinguish evidence-based medicine from traditional medicine, as the latter is charged with relying on unsystematic observations, medical intuition, pathophysiologic principles, and clinical experience [8].

While numerous techniques have been put in place to facilitate the systematic management, evaluation, and application of clinical data into evidence-based medical practice, the most distinctive technology is the hierarchy of evidence, a pre-graded ranking of experimental methodologies. Evidence-based medicine proponents strongly hold that the trustworthiness or validity of evidence is a function of the design of the study from which the evidence is obtained [9,10], and so the desire to use only the "best evidence from clinical research" in the management of individual patients [11] has resulted in elaborate classificatory schemes for ranking the value of different types of studies. Among the numerous published formulations [12,13], there is a consistent placement of randomised controlled trials or the systematic review of them at the top, retrospective studies well down the list, and clinical anecdotes are seen as providing little if any evidence for the value of intervention.

Evidence as accumulated data has been made widely and easily available to clinicians, educators, actuaries, and medical funding bodies by evolving information technologies such as electronic databases and systematic reviews of clinical trials. The political and professional capital of evidence-based medicine cannot be overstated, as this evidence-based practice is supposed to increase professional responsibility and accountability, improve patient care, and make managed care and medical research more cost effective by ensuring that only the most promising technologies are funded. The combined picture of evidence-based medicine as ethically driven to improve patient care, fiscally responsible, and technologically up-to-date likely drove the rapid integration of the movement into medicine, where fifteen years after the Evidence Based Medicine Working Group first formed, evidence-based medicine is now common parlance within health care. As a burgeoning institution, academic centres and journals dedicated to evidence-based medicine's advancement have been established with much fanfare, and the evidence-based movement has moved into numerous other fields, including nursing, public health, education and social work. The promise of the evidence-based movement to provide a systematic method for determining best practices has been so enthusiastically adopted in these fields that it is hardly surprising to find it generating attention as a promising new approach to bioethics in the form of "evidence-based ethics".

### Bioethics

Since its evolution into a distinct discipline, bioethics has typically employed the analytic methods of Anglo-American philosophy to answer the ethical questions that arise in health care. While the methods have diversified considerably from the deductive approaches of "applied ethics" that still typify the field to include casuist, contextualist, and reflexive methods, the field maintains a normative mandate. It is this self-understanding, and the worry that empirical approaches to bioethics waver on the fact/value distinction, that encouraged a significant antagonism toward empirical research in bioethics that is only beginning to subside [1]. Despite numerous contestations of this bifurcation [14], the descriptive and prescriptive sciences are still regarded as quite distinct entities.

Some commentators on the "empirical turn in bioethics" have regarded the interest in empirical research as representing a loss of confidence in the typical normative and analytic methods of bioethics [15]. While many describe those "typical" methods inaccurately – offering a "straw man" account of applied ethics where absolutely no empirical considerations are included in the deductive process of ethical deliberation, for example,  [16] – they are at least correct in recognising a felt ambivalence regarding the possibility of negotiating competing values in a pluralist society that respects difference. The technique of "evidence-based decision-making" offers what seems like a solution to this so-called "postmodern" problem, as it proposes to ground decisions in something concrete and universal, namely the evidence. The allure of evidence should not be underappreciated, as it is thought to be able to assist us in seeing past our habits, biases, and mistakes to decipher "best practices". The rapid ascendancy of the evidence-based movement, which started in medicine and quickly spread to other professional disciplines, speaks to the movement's enormous appeal. Even the popularity of the *CSI *television series – which depicts "evidence-based" police work *par excellence *– demonstrates how the stability, fairness, and truth of "the evidence" have captured our imagination. There are considerable difficulties with an "evidence-based" approach to bioethics, however, that require consideration.

### Evidence-based ethics

Evidence-based ethics has been defined in the literature as follows:

As in medical decisions based on evidence-based medicine, ethical decisions based on evidence-based ethics would involve conscientious and judicious use of the best evidence relevant to the care and prognosis of the patient to promote better informed and better justified ethical decision making [17].

What this actually entails in practice is somewhat vague, as there are numerous ways in which empirical research can inform ethical decision making, numerous types of evidence that are relevant to the care and prognosis of patients, and numerous measures of *best *evidence. What *is *clear, however, is evidence-based ethics' close methodological proximity to evidence-based *medicine*, as the language of "conscientious and judicious use of best evidence" is recognisably lifted from the early programmatic literature on evidence-based medicine [8]. The implications of this relationship are the focus of this paper, as evidence-based medicine offers a distinct accounting of the nature of evidence, what evidence counts, and what role the evidence plays in the decision-making process. Understanding evidence-based ethics requires comprehension of the evidence-based approach to medicine.

Jeremy Sugarman, a known supporter of evidence-base ethics, argues that in both medical and ethical investigations, "it is important to 'raise the bar' on what evidence is acceptable to determine the most effective approaches" [18]. In both cases, he argues, evidence derived from randomised trials has the most utility [18]. Sugarman's appeal for rigorous methods and his attention to experimental methodology recall the hierarchy of evidence's consistent privileging of randomised controlled trials and systematic review of these trials over less objective measures such as surveys or qualitative research. The founding of evidence based medicine by clinical epidemiologists and biostatisticians should explain this methodological privileging, as randomised controlled trials produce the clinical data required for health outcomes research.

Keeping in mind evidence-based ethics' subscription to the "evidence based" doctrine, it becomes apparent that the term "evidence-based ethics" has been misunderstood and misused by some of its alleged proponents. In Robert Jansen's paper, "Evidence-Based Ethics and the Regulation of Reproduction" [19], the author uses the term to mean the testing of ethical arguments, statements, and the background assumptions informing those arguments, by means of empirical research. Jansen argues that Canada's prohibitions on sex selection for human reproduction relies on the untested empirical claim that sex selection often leads to some index of family dysfunction. He finds it ironic that Canada insists on evidence-based approaches for medical services but not for the social restrictions on reproductive medicine proposed by the *Report of the Royal Commission on New Reproductive Technologies*, which, he claims, made determinations of "women's true interests" without properly surveying the relevant attitudes and behaviours exhibited by the public. Against what Jansen perceived as the Commission's "hijacking" of ethical questions and their treatment of empirically verifiable hypotheses about the social consequences of permissive policies as "self-evident moral truths", he recommends a publicly accountable empirical approach that encourages debate and the determination of facts.

Jansen's understanding of "evidence based ethics" seems to be no different from the empirical ethics already in circulation insofar as it serves to *inform *moral deliberation (and therefore does not introduce a new empirical/ethics relation) [20]. It is worth noting that even prior to the incipience of empirical ethics, empirical content always informed ethical deliberation, whether to determine the actual or probable consequences of actions for consequentialist reasoning or to specify the norms of deontological consideration. In bioethics, surveys or in-depth interviews that gauge patients' or clinicians' attitudes or behaviours often serve as the data that philosophically-trained bioethicists reflect on in order to draw moral conclusions [21].

The evidence-based medicine hierarchy of evidence's maligning of the very techniques that empirical ethics so often employs suggests dissimilarity between empirical ethics and evidence-based bioethics. The surveys and in-depth interviews that are commonly used to determine the attitudes and behaviours of patients, clinicians, or the general public regarding bioethical issues are less valued and are ranked lower than the carefully controlled and quantified evidence that is derived from randomised controlled trials and other more objective methods. This suggests evidence-based ethics to be a distinct moment within the "empirical turn in bioethics" rather than, as Pascall Borry and colleagues' historical account seems to suggests, more of the same [1].

The second sense in which evidence-based ethics is used is as "the necessary grounding of ethical decisions in the best available scientific evidence" [1]. Jon Tyson's [22] and Terri Major-Kincade and colleagues' [17] work on clinical determinations of whether or not to treat severely disabled premature newborns enlist this use of the term "evidence-based bioethics", which I read to be a more accurate interpretation of the term because of its consistency with the methods of evidence-based medicine. It has already been discussed that evidence-based medicine is typified by the systematic introduction of scientific proof in healthcare interventions. Health care practices are thought to surely improve by means of decision-making based on a careful appraisal of the best available scientific evidence [23]. Tyson's and Major-Kincade et al.'s work offers decision-making techniques for determining whether or not to treat the patient that rely almost exclusively on the projected survival and disability outcomes of these infants. Major-Kincade et al. even employ a controlled trial to demonstrate the efficacy of their educational curriculum for teaching evidence-based ethics to NICU residents.

Tyson describes evidence-based ethics as involving multiple considerations in its determination of what constitutes "*reasonable care*" that include: (i) the quality of evidence available; (ii) the identified benefits, hazards, and costs of treatment; and (iii) the values and preferences of the parent or surrogate. In Major-Kincade et al's complementary paper detailing the implementation of an evidence-based ethics educational intervention, the "evidence" was specified to mean mortality and disability outcomes for infants that receive intensive care.

Given that Tyson claims to appreciate that treatment decisions for extremely premature infants involve highly complex ethical issues and multiple considerations, it comes as a surprise when he proposes, in the end, an algorithm [24] for instances of "*mandatory*", "*unreasonable*", and "*optional*" treatment based entirely on the projected outcomes (that is, survival rates and disability-free years) for neonates of particular birth weights, gestational ages, and health conditions. Even the professed importance of considering the parents or surrogates' values and preferences is limited to situations where the infant's clinical indicators fit her into the category of "*optional*" treatment. While the description of the multiple considerations that go into evidence-based ethical decision-making sounded reasonably comprehensive at first glance, certain limitations on how evidence is understood, what constitutes a "benefit" or a "harm" and who determines and measures them, and even when the parents' values play in, all narrow the deliberative process to a decision based on projected outcomes and an imposed cost per value calculation of Quality Adjusted Life Years and Disability Adjusted Life Years relative to financial cost of treatment. Mandatory treatment, for example, occurs when there is "credible evidence that benefits outweigh burdens" [22], with is no mention of who determines these criteria and how they are measured. These determinations were formulated against the backdrop of standardised clinical protocols being simply assumed to be preferable, more transparent, and fairer than case-by-case decision-making. These assumptions will soon be demonstrated to be consistent with the "epidemiological and biostatistical ways of thinking" that the founders of evidence-based medicine so strongly promoted.

The feature of Tyson's and Major-Kincade et al.'s methods that truly exemplify an evidence-based approach is that rather than having a wide range of empirical evidence *inform *ethical decision making (as is seen in typical accounts of empirical ethics), their techniques use scientific evidence (narrowly construed) to *determine *right action. Against Sugarman's claim that "empirical research [into bioethics] will not answer the ought question of bioethics" [4], evidence-based ethics seems to do just that. The slide from "is" to "ought" has already been noticed in evidence-based medicine. While the *is *of evidence-based medicine is that science is producing new and better ways of predicting, detecting and treating disease than were once even imaginable, the *ought *is that its advocates believe that clinicians ought to be responsible for keeping up to date with these advances and ought to be prepared to offer them to patients. Brian Haynes, one of the founders of the movement who has noticeably tempered his proclamations in recent years about the transformative ability of evidence-based medicine, has noted that "evidence-based medicine has taken on the tones of a moral imperative [even though] it is premature to get very preachy about the ought of evidence-based medicine" [25]. Another similarity between evidence-based medicine and evidence-based ethics is that the scope of "scientific evidence" is narrowed to exclude most forms of social scientific and qualitative evidence and is limited instead almost exclusively to medical outcomes, which is the evidence of choice according to the methodological hierarchies of evidence-based medicine.

The evidence-based doctrine problematically assumes that the presence of reliable evidence ensures that better decisions will be made. Medical decision-making, however, draws upon a broad spectrum of knowledge (or multiple dimensions of evidence), including scientific evidence, personal experience, personal values, economic and political considerations, and philosophical principles. It is not always clear how practitioners integrate these factors into a final decision, but what *is *clear is that medicine can never be entirely free of value judgments [26]. Normative content seems to enter at all levels of decision-making, even in the production and presentation of the scientific evidence that is supposed to univocally inform evidence-based decisions [27]. The very notion of evidence and the boundaries of what counts as evidence is a social construct, as evidence is always the product of a socially produced question. Even "evidence-based" is a normative concept.

In Tyson's attention to systematic measures and formulaic approaches, he glosses over the value judgments that go into the evaluation of "*reasonable*" and "*unreasonable*" actions. He similarly takes as "given" the implicit normativity in his "medical cost relative to value" formula for deciding how to use limited health care resources. In his accounting, the cost utility of neonatal intensive care is expressed as the cost per quality-adjusted life-year (QALY) gained as a result of neonatal intensive care. The life-years gained are then reduced according to the number of disabled survivors and the severity of those disabilities. While Tyson seems to think that deferral to measurement is transparent and fair – presumably because the life circumstances of individual families do not bias the assessment – the values implicit in these measures go unchecked. Even his recognition of "the fact that it is difficult to know how to adjust appropriately for disability and disease, in part because quality of life in the presence of handicaps and chronic illnesses may be rated higher by those affected than by other persons" does not seem to deter him from formulating an evidence-based decision-making algorithm and assuming the justice of measurement in general.

By this account of evidence-based ethics, one might ask how evidence-based ethics differs from evidence-based medicine, as both involve making health care decisions based on the best evidence, where evidence is narrowly defined as having to do with systematic observations from certain types of scientific research. Alternatively, one might question whether evidence-based ethics represents a misappropriation of the word "ethics" [28].

### "Evidence based" approaches and practices

While "evidence-based" is typically read in medicine and other life and social sciences as the empirically-adequate standard of reasonable practice and as a means for increasing certainty, the evidence-based movement in fact gains consensus by *displacing *normative discourse with aggregate or statistically-derived empirical evidence as the "bottom line". The techniques invoked in the name of "evidence-based" decision-making require a positivistic reliance on "the evidence" in its epistemological promise to ascertain truth or certainty by examination of the evidence. These techniques act to obscure the multiple and complex considerations that unavoidably go into health care decisions at both the micro- and macro- level and allows for the promotion of particular political agendas and interests under the guise of "better science" [29].

Despite the promise to revolutionise medicine and the language of "new paradigms", the term "evidence based medicine" has a ring of obviousness to it that makes it difficult to argue against. Few physicians, one suspects, would be willing to assert that they do not attempt to base their clinical decision-making on available evidence. Scientific progress, in fact, is popularly understood to have been motivated by the evidence-based practices of innovative scientists. Rejecting the dogma and superstition that pervaded their historical moment, these innovators let the evidence, as gathered through unbiased and careful experimentation, dictate their scientific practices, beliefs, and theories.

Yet the seeming obviousness of evidence-based medicine is suspect. Post-positivist philosophies of science over the past half century have contested the popular understanding of observational evidence as value-free, pretheoretical, self-apparent, and therefore sufficient to verify or at least falsify scientific hypotheses [30]. The social nature of science is thought to involve considerable normative content in its knowledge producing activities, and these values are not excised in the context of justification. Feminist epistemologists of science, such as Helen Longino [31] and Lynne Hankinson Nelson [32], have called for explicit recognition and critical appraisal of these values, as techniques that presume the value neutrality of science in fact distort scientific practice. The same concern arises in medicine, where evidence-based medicine only *seems *common sense because it has been stripped of the social context of medical practice in its professed deferral to only "the evidence". In an age where the institutional power of medicine is suspect, a model that represents biomedicine's power as disinterested (or merely "scientific") should give pause. Keith Denny reads evidence-based medicine as a discourse that resists contemporary challenges to established medical authority [33]. While evidence-based medicine appears to question the authority of individual physicians, it works instead to reinforce that authority through its regulation. Furthermore, evidence-based medicine does *not *question the institutional authority of medicine within society, the way healthcare dollars are allocated for the necessary clinical research, and what role the pharmaceutical industry plays in setting the research agenda.

Evidence-based practices maintain the distinct ability to sidestep value differences and political disputes by appealing to the evidence as the bottom line. This move is positivist in its elimination of culture, contexts, and the subjects of knowledge production from consideration. It is also attractive in an age of moral pluralism. This conceptual linking of methods of abstraction to ascertain truth and progressive politics is reminiscent of the radical politics of early logical positivists like Otto Neurath. In post-war Germany, an epistemological system that avoided the pitfalls of fascism and successfully unified systems of science and thought was perceived to be a progressive step. Only later did "unity of science" theses come to be seen as imperialist and assimilationist and rejected by innovative thinkers in favour of "disunity" and "pluralist" post-modern positions [34].

In health care justice and policy, we see appeals by liberal thinkers to allegedly neutral markers like "species normal functioning". We know, of course, that these measures are not neutral, as people with disabilities and chronic illnesses and elderly people consistently fare poorly in this political calculus. Popular thinking holds, however, that if it is neutrality that is desired, numbers are the pinnacle. In this age of the ascendancy of health outcomes research, where statistical analysis dominates health policy decisionmaking, "evidence" is tantamount to *measure *and not *meaning*.

Statistical inference is pursued precisely for its superficiality and its ability to measure broad rather than individual experience. It was its ability to isolate more general variables and phenomena that permit more open and egalitarian debate about social questions that caught the attention of liberals and would-be reformers such as Neurath and Auguste Comte. Yet generalisations and standards contain implicit socially framed and mediated values with a range of implications that *can *order and enhance, but also tyrannise, aspects of our lives. The success of generalisation is achieved at the expense of contingent and contextual knowledge that needs to be filtered out [35].

Evidence-based decision-making faces inherent limitations insofar as only certain kinds of experience can be quantified and only certain questions explored [26]. Data-driven approaches to patient care have been argued to narrow our ability to effect actions in clinical encounters, as statements of averaged probability become unquestioned laws of possibilities [36]. They limit appreciation of the subtleties and exceptions that characterise all efforts to diagnose and treat illness and displace the critical and vast source of information for treatment, diagnosis and meaningful management of illness that is found in human interaction. Furthermore, evidence-based decision-making ignores the contingency of medical knowledge. While efforts to capture such encounters in aggregate terms have become increasingly sophisticated and thorough, the limitations just mentioned are part and parcel of epidemiological methods and simply cannot be overcome.

We ought to be attentive to the historical moment in which we find ourselves, when the challenges and questions raised by the fragmentation of the medical subject and its recreation as an average probability seem "obvious". In his historical account of the rise of managed care, Gary Belkin situates managed care not as an inevitable response to cost control and economic inefficiency, but within a history of appeals to standardised and ostensible objective measures and models of human behaviour to resolve contentious issues in complex and modern capitalist democratic societies [37]. Evidence-based medicine and evidence-based ethics similarly resolve normative questions and policy issues, such as whether or not to treat a newborn with severe disabilities, by transforming them into problems of measurement. Interestingly, the appeals to technical formulas and standardised information satisfies the strong wish that we have for open, reliable, and presumably objective methods for the resolution of controversy.

Because evidence-based medicine is largely an effort to manage the unruly social world (in which medicine is practiced) via objective scientific procedure, the movement appears to be the latest expression of "scientism", modernity's rationalist dream that science can produce the knowledge required to emancipate us from scarcity, ignorance, and error. However, such efforts tend to disguise political interests in the authority of so-called "scientific evidence". The configuration of policy considerations and clinical standards into questions of evidence conveniently transforms normative questions into technical ones. Political issues are not resolved, but merely disguised in technocratic consideration and language. Thus the goals of medicine and other normative considerations lie just below the surface of these evidentiary questions, and evidence becomes an instrument of, rather than a substitute for, politics.

To illustrate this, consider that the outcomes movement has been invoked to support political efforts to increasingly privatise American health care [38], as the valuing of standardised measures, aggregate behaviour, and radically fragmented medical knowledge supports the logic of the medical marketplace. Because evidence-based medicine legitimates the distillation of medical truth outside of the clinical encounter, where statistical information is privileged over the physician's clinical judgment in clinical decisionmaking, the rationale of a healthcare marketplace populated by independent and rational buyers and sellers is validated. It is in the name of "better science" that particular economic interests can be furthered. The enthusiasm for standardised measures in clinical practice, consistency among professionals in therapeutic interventions, and gold standards of clinical science reflect a medical logic that prefers abstracted measure over individualised history and pathology. Medical authority is, therefore, no longer framed in scientific discourse but in late 20^th^/early-21^st ^century capitalist discourse with its ideological extolling of the importance of "information" – a move that co-opts demands for democracy and holism in medicine [33]. This is done by appeal to "the evidence" – the unbiased bottom line. Evidence serves as a tool to maintain power by attempting to ignore the conflict of norms at play in politically contentious issues. Habermas has argued that the separation of the technical and the political is an instructive mark of modernity [39]. This removal of normative content from the ideological apparatuses has the dangerous effect of depoliticising the organization of social life and therefore justifying its institutions by rendering them functional within a system of supposedly technically necessary activity.

Much like positivism threatened ethics by rendering it "senseless", an "evidence based" approach proposes to make moral deliberation redundant as it offers a method to resolve ethical and political questions about healthcare spending and practice by appeal to technical measure. The normative issues therefore get co-opted by supposedly neutral technique. An "evidence based" ethics would therefore threaten bioethics' normative mandate.

## Summary

In medicine, bioethicists are typically attuned to the multiple dimensions of the illness experience that eludes quantification and measurement. Science, according to medical humanists, is just one layer of description of the phenomenological world. Evidence-based medicine's reliance on scientific evidence has been criticised for mischaracterising modern health care's constitution by diverse academic traditions and knowledges – including the humanities, social sciences, and the pure and applied sciences – that rely on equally diverse notions of evidence [40]. While bioethicists attend to the normative features of medical decision-making, evidence-based ethics suggests a moment of inattentiveness to the normativity of *moral *decision-making. Recognition of the plurality of values and meanings in operation complicates our use of moral and ethical terms and categories; however, the quick turn to various truth-producing strategies labelled "empirical" that has taken place warrants careful consideration. While the "empirical turn" in bioethics signals a need for reconsideration of the methods used for moral evaluation and resolution, the options should not include obscuring normative content by seemingly neutral technical measure.

## Competing interests

The author(s) declare that they have no competing interests.

## Pre-publication history

The pre-publication history for this paper can be accessed here:


